# Efficacy of a novel topical combination of esafoxolaner, eprinomectin and praziquantel against fleas in cats, under field conditions

**DOI:** 10.1051/parasite/2021018

**Published:** 2021-04-02

**Authors:** Eric Tielemans, Tomoko Otsuki, Tara Cheesman, Fiona Selmes, Anthony Pfefferkorn, Joe Prullage

**Affiliations:** 1 Boehringer-Ingelheim Animal Health 29 Avenue Tony Garnier 69007 Lyon France; 2 Boehringer-Ingelheim Animal Health, Missouri Research Center 6498 Jade Rd. Fulton 65251 MO USA; 3 Boehringer Ingelheim Animal Health Japan Co. Ltd. ThinkPark Tower, 2-1-1 Osaki Shinagawa-Ku 141-6017 Tokyo Japan; 4 Boehringer Ingelheim Animal Health (Pty) Ltd. 78 Waterloo Rd. North Ryde 2113 NSW Australia

**Keywords:** Cat, *Ctenocephalides felis*, Esafoxolaner, Field efficacy, Safety

## Abstract

Esafoxolaner is a purified afoxolaner enantiomer with insecticidal and acaricidal properties. It is combined with eprinomectin and praziquantel, nematodicidal and cestodicidal compounds, in a novel topical endectoparasiticide formulation for cats. This novel formulation was tested in four field studies, in the United States, Europe, Japan and Australia. In all studies, naturally flea-infested domestic cats were treated with the novel formulation at the label dose and conditions of use. The main objective, identical in the four studies, was to assess efficacy on fleas, based on comparison of mean number of fleas found on infested cats before and one month after treatment. Tolerance to the product was also evaluated in the four studies. Otherwise, the studies had some differences in their design and secondary objectives, for example testing for a reduction in flea infestation-related cutaneous signs, testing of one treatment or of three monthly treatments, and use of a positive control group. In the four studies, a total of 307 cats were treated with the novel formulation. The reduction of fleas one month after treatment was 97.7%, 98.8%, 100% and 99.7% in the United States, Europe, Japan and Australia, respectively. There were no significant health abnormalities attributed to treatment in any of the studies.

## Abstract (fr)

L’esafoxolaner est un énantiomère d’afoxolaner purifié aux propriétés insecticides et acaricides, et il est associé à l’éprinomectine et au praziquantel, des composés nématodicides et cestodicides, dans une nouvelle formulation d’endectoparasiticide topique pour chats. Cette nouvelle formulation a été testée dans quatre études sur le terrain, aux États-Unis, en Europe, au Japon et en Australie. Dans toutes les études, des chats domestiques naturellement infestés de puces ont été traités avec la nouvelle formulation à la dose et aux conditions d’utilisation indiquées sur l’étiquette. L’objectif principal, identique dans les quatre études, était d’évaluer l’efficacité contre les puces, sur la base de la comparaison du nombre moyen de puces trouvées sur des chats infestés avant et un mois après le traitement. La tolérance à l’application du produit a également été évaluée dans les quatre études. Sinon, les études présentaient des différences dans leur conception et leurs objectifs secondaires, par exemple test de réduction des signes cutanés liés à l’infestation par les puces, test d’un traitement ou de trois traitements mensuels, utilisation d’un groupe témoin positif. Dans les quatre études, un total de 307 chats ont été traités avec la nouvelle formulation. La réduction du nombre de puces un mois après le traitement était de 97,7 %, 98,8 %, 100 % et 99,7 % aux États-Unis, en Europe, au Japon et en Australie, respectivement. Aucune anomalie de santé significative n’a été attribuée au traitement dans aucune des études.

## Introduction

Infestations with ectoparasites are amongst the most frequent parasitic disorders in carnivores worldwide, and fleas, ticks and ear mites are the most common of these [4, 22, 23]. The cat flea, *Ctenocephalides felis,* is widespread and is the predominant flea species found in both cats and dogs [3, 20, 25]. Flea bites induce inflammatory skin reactions and can result in alopecia, erythema, or dermatitis. Heavy infestations can cause anaemia [5, 11, 26, 27]. Flea bite hypersensitivity, also called flea allergy dermatitis (FAD), is one of the most common dermatological conditions in companion animal veterinary medicine, and includes symptoms such as pruritus, miliary dermatitis, crusts, and alopecia [1, 5, 8, 24, 29]. *Ctenocephalides felis* can also transmit zoonotic agents such as *Rickettsia felis*, the agent of flea-borne spotted fever, or *Bartonella henselae*, the agent of cat scratch disease. Fleas are the intermediate host for *Dipylidium caninum* [1, 5, 7, 10, 16, 18, 19, 21].

A novel topical combination of esafoxolaner, eprinomectin and praziquantel has been developed with the aim of offering a wide spectrum of antiparasitic activity. Afoxolaner is a racemic mixture and esafoxolaner is the active purified (S)-enantiomer. Afoxolaner has been proven effective against adult fleas and flea egg production in dogs [4, 15, 17].

This novel topical combination has demonstrated efficacy against adult and immature *C. felis* flea stages in cats under laboratory conditions [27].

This article describes four studies performed to assess the efficacy and the tolerance of this novel formulation under field conditions in several regions of the world with different climates for the treatment and control of natural *C. felis* flea infestations when administered topically to cats.

## Materials and methods

### Ethics

The four study protocols had been reviewed and approved by the Sponsor’s Institutional Animal Care and Use Committee, and a license had been obtained from the local authorities for each trial in each country.

### Study designs

The four studies were designed in accordance with the “World Association for the Advancement of Veterinary Parasitology (WAAVP) guidelines for evaluating the efficacy of parasiticides for the treatment, prevention and control of flea and tick infestation on dogs and cats” [20]. Specific local guidelines were also applied, for the European Union (EU): “Guideline for the Testing and Evaluation of the Efficacy of Antiparasitic Substances for the Treatment and Prevention of Tick and Flea Infestation in Dogs and Cats”, EMEA/CVMP/EWP/005/2000-Rev.3; for Australia: “Preamble for the WAAVP guideline for fleas and ticks on dogs and cats version 1 (1 July 2014)”.

The four studies were conducted in accordance with Good Clinical Practices as described in “International Cooperation on Harmonization of Technical Requirements for Registration of Veterinary Medicinal Products (VICH) guideline GL9”.

The studies were carried out on domestic cats under the responsibility of a veterinarian, in different regions of the world, i.e. 11 sites in the United States: Florida (3), Louisiana (2), Missouri (2), Oregon (1), Tennessee (1), Georgia (1), and Mississippi (1); 16 sites in Europe: France (9), Portugal (1), Germany (3), Hungary (1), Bulgaria (1), Romania (1); 11 sites in Japan in Kanto, Chubu and Kyushu: Tokyo (2), Gunma (3), Nagano (1), Fukuoka (1), Kumamoto (3) and Okinawa (1); and 2 regions in Australia: Queensland (2), and New South Wales (2).

The primary objective of the four studies was to evaluate efficacy of the novel formulation against fleas one month after an application. Nevertheless, the four studies had different contexts, secondary objectives and designs, as summarized in Table 1.

**Table 1:** Study designs.

Field trial	Objective(s)	Location	Date	Positive control	Ratio IVP/PCP	Primary variables of efficacy
USA	Flea efficacy & safety for 3 months, reduction of flea infestation-related cutaneous signs	Southeast, Midwest, Western USA	Apr 2017 – Nov 2018	Selamectin	2/1	Flea reduction at 1 month for sentinel cats
EU	Flea efficacy & safety for 1 month, reduction of flea infestation-related cutaneous signs	Western, Central, Eastern Europe	Apr 2017 – Oct 2017	Fipronil, (S)-methoprene	2/1	Weekly flea reduction for sentinel cats for 1 month, non-inferiority versus positive control
Japan	Flea efficacy & safety for 1 month	6 prefectures in Kanto, Chubu and Kyushu	Jul 2017 – Jan 2018	Selamectin	1/1	Flea reduction at 2 days and 1 month (all cats), non-inferiority versus positive control
Australia	Flea efficacy & safety for 1 month	NSW, QLD	Jan 2019 – Mar 2019	NA	NA	Flea reduction at 1 month for sentinel cats

For inclusion, the cats were naturally infested with at least five fleas (at least six fleas in Australia). Neither the cat nor the environment had been treated with any ectoparasiticide compound within four weeks of inclusion. Cats were randomly allocated to the novel formulation or the positive control group based on order of presentation at the site in a 2:1 ratio (US and EU) or 1:1 ratio (Japan), except in Australia where no control group was used. In the USA, EU and Australia, one cat with appropriate flea infestation, the sentinel cat, was selected per household for efficacy and tolerance evaluation. When applicable, the other cat(s) of the same household were treated with the same drug as the sentinel cat and only evaluated for tolerance. In the EU (all cats) and the USA (sentinel cats), study cats affected by flea infestation-related cutaneous disorders were evaluated for reductions of these signs. In Japan, in multi-cat households, each cat was independently allocated, treated and evaluated for efficacy and tolerance.

Most study activities (flea infestation evaluations, treatments, and physical examinations) were performed at the veterinary practices, except day-to-day owner observations. The study activity schedules are summarized in Table 2.

**Table 2:** Study schedules.

Study	Day 0	Day 2	Day 7	Day 14	Day 21	Day 30	Day 60	Day 90
USA (sentinels^a^)	RC, T	–	–	–	–	RC, T	RC, T	RC
EU (sentinels^b^)	RC, T	–	RC	RC	RC	RC	–	–
Japan (all cats)	isC, T	RC	–	–	–	RC	–	–
Australia (all cats)	RC, T	–	–	–	–	RC	–	–

RC = flea removal and count; T = Treatment; isC = *in situ* flea thumb count (without flea removal).

a In the USA, non-sentinel cats were only presented on Day 0 and Day 90 for physical examination and cutaneous assessment, and were treated at home by the owners on Days 30 and 60.

b In the EU, non-sentinel cats were only presented on Day 0 and Day 30 for physical examination and cutaneous assessment.

Physical examinations for tolerance evaluations were performed at all visits (except in Australia).

The flea efficacy assessment was based on comparison of the number of live fleas found before treatment to the number of live fleas found after treatment, at the scheduled visits.

In Australia, blinding was not applicable as no control group was used; otherwise, all personnel collecting efficacy, tolerance or flea-infestation cutaneous signs data were blinded to treatment. Owners were blinded to treatment in the EU and Japan. In the USA, the owners were not blinded as they administered the treatments.

Any drug that could influence the safety and/or efficacy assessments was not allowed. Any concurrent medication that could positively influence the resolution of cutaneous signs (e.g. corticosteroids, immunomodulators, dermatological topical substances, or medicated shampoos) were allowed for animal welfare purposes. Nevertheless such use excluded the animal from evaluation of reduction of flea infestation-related cutaneous signs.

### Animals

All animals were domestic cats of various ages (8 weeks to >8 years), bodyweights (minimum 0.8 kg), sex and reproductive statuses (neutered or intact). Cats were predominantly mixed Domestic Short, Medium, and Long-hair breeds, but some pure-bred cats were also included, e.g. Siamese, Maine Coon, Persian, American and British Shorthair, Manx, Tonkinese, Norwegian forest cats, Birman, and Angora.

Cats lived indoors, outdoors, or had both indoor and outdoor access; no housing restrictions were applied.

### Flea strains and species

Fleas were naturally acquired. Samples of collected fleas were examined morphologically in the EU and Japan for species identification. In Japan, 100% of the fleas were *Ctenocephalides felis*; in the EU 96.4% were *Ctenocephalides felis*, 1.5% *Ctenocephalides canis,* 0.8% *Archaeopsylla erinacei*, and the remaining species (<0.5% each) were *Nosopsyllus fasciatus, Spilopsyllus cuniculi, Ceratophyllus* sp*.,* and *Xenopsylla* sp*.*

### Treatment

In the EU, Japan and Australia, cats were treated once on Day 0 by qualified unblinded personnel, after flea and physical evaluations. In the USA, cats were treated on Days 0, 30 and 60 by the owner in the veterinary practice after flea count and physical evaluations (or at home for the non-sentinel cats on Days 30 and 60). For each treatment, cats assigned to the novel formulation treated group received a topical application at the recommended label dose of 0.3 mL (for cats weighing 0.8 to <2.5 kg), or 0.9 mL (for cats weighing 2.5 to <7.5 kg), which delivered 1.44–4.5 mg/kg esafoxolaner, 0.48–1.50 mg/kg eprinomectin, and 10.0–31.1 mg/kg praziquantel. The treatments were applied in one spot directly on the skin, after parting the hair, in the midline of the neck between the base of the skull and the shoulder blades.

In the USA and Japan, cats assigned to the control groups were treated with selamectin 6% (Revolution^®^ for cats), and in the EU with fipronil 10%/(S)-methoprene 12% (Frontline Combo^®^ Spot-on cat), each time at the recommended dose and conditions of use.

### Flea counts

Flea counts were performed at schedules defined in Table 2, by systematically combing all parts of the cat using a fine-tooth flea comb for at least 5 min, except in Japan on Day 0, when thumb counts were performed and fleas not collected.

Overall, 307 cats were evaluated for efficacy of the novel formulation, one month after treatment.

### Tolerance

In the four studies, owners were requested to observe their animals daily and report any abnormality to the clinic. At each scheduled visit, the Investigator performed a physical examination, which together with a consideration of any adverse event reported by owners (resulting or not in an unplanned veterinary consultation, veterinary care, concurrent medication, etc) was taken into account for evaluation of tolerance. Relationship to treatment for all adverse reactions and abnormalities was evaluated by the Investigator.

Overall, 702 cats were evaluated at least once for safety of the novel formulation, and a total of 2512 observations were made by the Investigators (inclusive of all studies and all evaluation time-points). This number is higher than the 307 cats assessed for flea efficacy due to multi-cat households in the USA and in Europe, where only one of the treated cats, the “sentinel-cat” was included in flea counts.

### Flea infestation-related cutaneous signs

Reduction of flea infestation-related cutaneous signs such as alopecia, miliary dermatitis, excoriation, scaling, and erythema were evaluated in the USA on sentinel cats during the three months of the study, and in Europe on all cats during the month of the study.

### Statistical analyses

Only sentinel cats were included in the efficacy evaluation (except in Japan, where all cats in multi-cat households were individual experimental units). Total live flea counts were transformed to the natural logarithm of (count + 1) for analysis and calculation of the geometric means. The percent efficacy was calculated using geometric means using the formula 100 × ([*B* − *T*)/*B*], where *B* = mean of the Day 0 (baseline) visit count, and *T* = mean of the appropriate visit day count. The geometric mean was calculated by taking the anti-logarithm of the average of the log-counts and then subtracting 1, or was computed by taking the anti-logarithm of the least square mean −1 from the analysis model.

## Results

### Efficacy against fleas

Efficacy against fleas in the four studies is summarized in Table 3. The calculated efficacy, one month after a single topical application of the novel formulation was 97.7%, 98.8%, 100% and 99.7% in the USA, Europe, Japan and Australia, respectively (*p* < 0.0001 for the four studies).

**Table 3:** Efficacy results.

		% Efficacy in the different field studies						
		Day 2	Day 7	Day 14	Day 21	Day 30	Day 60	Day 90
#1 USA	Novel formulation^a^	–	–	–	–	97.7	99.6	99.9
#1 USA	Revolution^®^ b	–	–	–	–	82.8	91.7	94.2
#2 EU	Novel formulation^a^	–	98.3	97.5	99	98.8	–	–
#2 EU	Frontline Combo^®^ c	–	86.4	89.6	84.6	86.5	–	–
#3 Japan	Novel formulation^a^	99.0	–	–	–	100.0	–	–
#3 Japan	Revolution^®^ b	98.3	–	–	–	100.0	–	–
#4 Australia	Novel formulation^a^	–	–	–	–	99.7	–	–

*p* < 0.0001 for all values at Day 30.

a Esafoxolaner, eprinomectin, praziquantel.

b Selamectin.

c Fipronil, (S)-methoprene.

The USA and the EU field studies each included a control group, using a reference ectoparasiticide product. Both control products provided a good level of flea control after one treatment; however, both below the 90% threshold. Topical selamectin (Revolution™) provided an average of 82.8% flea reduction, and fipronil/(S)-methoprene (Frontline Combo^®^) 86.5% one month after a single treatment in the USA and EU, respectively. A second and a third treatment were necessary in the US field study for selamectin to exceed the 90% threshold as efficacy increased to 91.7% at two months, and 94.2% at three months (there was no repeated treatment scheduled in the EU field trial).

### Tolerance

In the four trials, a total of 702 cats (including non-sentinel cats) received a total of 1173 applications of the novel formulation and were observed 2512 times by Investigators and daily by owners for 1 month, or 3 months in the USA. Health abnormalities of possible or unknown relationship to treatment as reported by owners or evaluated by the Investigator (excluding adverse reactions deemed unrelated to treatment) were mild, infrequent, and self-limiting. They included emesis (17 observations), application site reaction/pruritus (9), anorexia (9), hypersalivation (8), apathy (7), loose faeces (5), malodorous coat (2), and hyperthermia (2).

### Reduction of flea infestation-related cutaneous signs

In both the USA and EU trials, animals affected by flea infestation-related cutaneous signs had markedly improved at the end of the study, including both the novel formulation treated animals and the comparator product treated animals.

## Discussion

In these four studies, the novel topical formulation of esafoxolaner, eprinomectin and praziquantel demonstrated a high level of efficacy for the treatment and control of fleas in field conditions for at least 1 month, and in several major regions and climates around the world. Flea infestation related-cutaneous signs were markedly improved after one month. Tolerance to the novel formulation was proven excellent by several hundred applications to cats from a wide variety of living conditions, ages, both sexes, and breeds.

These field studies confirm the efficacy results obtained in four laboratory studies testing efficacy of the same product against adult and immature fleas [27]. In addition to controlled laboratory studies with a standardised and accurate level of infestation, it is important to test new products under field conditions, where infestations offer different challenges than in laboratory settings and can significantly vary in intensity and duration. In the field, pets are predominantly infested by fleas emerging from their environment [6]. Fleas are extremely prolific: a female during its adult life of two to four weeks lays an average of 20–30 eggs daily that fall off the host. These eggs need two to several weeks to develop into new adult fleas [5, 11], which can result in heavy environmental infestations when the animals and the environment are left untreated [12, 13]. Consequently, sustained efficacy of the flea product and inhibition of flea egg production or immature development are necessary to provide optimal control. Several treatments may be necessary to break the flea life cycle and to decrease the environmental flea burden [14]. In these four studies, a single application of the novel formulation resulted in a high level of flea control during the first month after application, with reductions of at least 97.7%, above the commonly accepted threshold of 90% [20]. The comparator products used in the USA and EU studies, selamectin and fipronil/(S)-methoprene respectively, did not achieve the commonly accepted 90% efficacy threshold after a single topical application. An improvement was seen in the USA study following the second and the third application of selamectin (such information was not available for fipronil/(S)-methoprene, as it was only applied once in the EU study), which demonstrated the efficacy of selamectin following repeated treatments. These data show an advantage of the novel esafoxolaner, eprinomectin and praziquantel formulation against both comparator products after one month, which was not statistically assessed due to the non-inferiority type design of the studies.

The objective of this novel association of esafoxolaner, eprinomectin and praziquantel is to offer a high level of efficacy against a broad spectrum of ecto- and endoparasites of cats. The control of multiple and various concurrent parasitic infestations by a range of cat parasites is important for cats, but also for public health [2, 9, 28].

In addition to a high level of efficacy and safety, owner compliance with the administration regime and ease of administration to the cat are important features for successful control of fleas. The simple conditions of use of this product should guarantee a high level of compliance.

## Competing interest

The work reported herein was funded by Boehringer-Ingelheim. The authors are current employees of Boehringer-Ingelheim. Other than that, the authors declare no conflict of interest. This document is provided for scientific purposes only. Any reference to a brand or trademark herein is for information purposes only and is not intended for any commercial purposes or to dilute the rights of the respective owners of the brand(s) or trademark(s).
